# Fracture risk assessment in patients with ileal urinary diversion after radical cystectomy: a comprehensive evaluation integrating bone mineral density, trabecular bone score, and FRAX®

**DOI:** 10.1007/s11657-026-01685-x

**Published:** 2026-03-11

**Authors:** Arturo Domínguez, Enrique Casado, Jesús Muñoz-Rodríguez, Joan Prats, María Laura Grubicy, Núria Masip, Cristina Piqué, Clara Centeno, Teresa Bonfill, Diana Fuertes, Xavier Serra-Aracil

**Affiliations:** 1 Department of Urology, Parc Taulí Hospital Universitari, Institut d’Investigació i Innovació Parc Taulí (I3PT-CERCA), Universitat Autònoma de Barcelona, Departament de Cirurgia, Sabadell, Spain; 2 Department of Rheumatology, Parc Taulí Hospital Universitari, Institutd’Investigació i Innovació Parc Taulí (I3PT-CERCA), Universitat Autònoma de Barcelona, Sabadell, Spain; 3 CETIR ASCIRES, C/Viladomat 299, Barcelona, Spain; 4 Department of Medical Oncology, Parc Taulí Hospital Universitari, Institut d’Investigació i Innovació Parc Taulí (I3PT-CERCA), Universitat Autònoma de Barcelona, Sabadell, Spain; 5 Research Support Unit, Parc Taulí Hospital Universitari, Institut d’Investigació i Innovació Parc Taulí (I3PT-CERCA), Universitat Autònoma de Barcelona, Sabadell, Spain; 6 Department of Surgery, Parc Taulí Hospital Universitari, Institut d’Investigació i Innovació Parc Taulí (I3PT-CERCA), Universitat Autònoma de Barcelona, Departament de Cirurgia, Sabadell, Spain

**Keywords:** Radical cystectomy, Urinary diversion, Bone mineral density, Trabecular bone score, FRAX, Metabolic acidosis

## Abstract

***Summary*:**

We conducted a comprehensive fracture risk assessment in 112 men with urinary diversion (UD) after radical cystectomy, integrating bone mineral density (BMD), trabecular bone score (TBS), and FRAX®. A substantial proportion met the diagnostic criteria for osteoporosis or had lower TBS values. These findings support routine skeletal assessment during follow-up.

**Purpose:**

Patients undergoing radical cystectomy with UD face an increased risk of fracture; however, this aspect remains poorly investigated. We aimed to provide an integrated evaluation of skeletal health and fracture risk in men with UD.

**Methods:**

We conducted a cross-sectional study of 112 men (mean age 70.6 ± 7.98 years; range 50–83) with ileal UD. BMD was measured using DXA, TBS was obtained from lumbar spine DXA images, and 10-year fracture risk was estimated using FRAX®. Logistic regression was used to identify predictors of osteoporosis and lower TBS values.

**Results:**

Osteoporosis was identified in 32.1% of patients, and 40.2% were classified in the lowest TBS category (TBS < 1.23). Only 12.5% had normal results for both BMD and TBS. Median FRAX-MOF was 3.9% (IQR 2.7–6.05) without BMD, 5.3% (2.98–7.82) with BMD, and 5.8% (3.4–9.5) when adjusted for TBS. Median FRAX-HIP was 1.35% (0.7–2.78), 1.8% (0.9–4.55) with BMD, and 2% (1–5.2) with TBS adjustment. Age (per year, OR = 1.11, 95% CI 1.02–1.20; *p* = 0.012) and BMI (per kg/m^2^, OR = 0.73, 95% CI 0.62–0.86; *p* < 0.001) were independently associated with osteoporosis, while serum creatinine (per mg/dL, OR = 2.11, 95% CI 1.08–4.11; *p* = 0.028), BMI (per kg/m^2^, OR = 0.91, 95% CI 0.83–0.99; *p* = 0.041), and prior fracture (OR = 5.23, 95% CI 2.08–13.13; *p* < 0.001) were associated with TBS < 1.23. Metabolic acidosis was not associated with adverse skeletal outcomes or higher FRAX estimates.

**Conclusions:**

Men with ileal UD showed reduced bone mass and a notable proportion of low TBS values, consistent with increased skeletal fragility and fracture risk. Structured bone health assessment should be considered during routine follow-up.

**Supplementary Information:**

The online version contains supplementary material available at 10.1007/s11657-026-01685-x.

## Introduction

For decades, radical cystectomy (RC) with ileal urinary diversion (UD) has been the standard treatment for muscle-invasive bladder cancer. Although this procedure improves cancer-specific survival, it may result in long-term metabolic complications that negatively impact skeletal health and increase the risk of fracture [1, 2]. Recent studies indicate that patients with UD have a 21–48% higher risk of fragility fractures than the general population [3, 4]. Despite this evidence, bone health evaluation in these patients remains uncommon, and key pathophysiological drivers are still poorly defined.

Dual-energy X-ray absorptiometry (DXA) is the standard method for assessing bone mineral density (BMD) and identifying individuals at risk of fracture. To date, only three studies have examined the prevalence of osteoporosis in UD-patients, reporting highly variable rates ranging from 0 to 36% due to differences in methodology and sample size [5–8]. However, BMD alone does not capture all aspects of skeletal fragility. Trabecular bone score (TBS) provides a validated gray-level textural index derived from lumbar spine DXA images that is associated with fracture risk independently of BMD. [9]. Beyond imaging, the Fracture Risk Assessment (FRAX®) tool enables clinicians to estimate the 10-year probability of major osteoporotic and hip fractures by integrating clinical risk factors, with or without BMD or TBS [10, 11]. To date, both TBS and FRAX® remain largely unexplored in patients with UD, leaving an important gap in the comprehensive assessment of fracture risk in this vulnerable group.

Several mechanisms may explain the skeletal deterioration associated with UD. Metabolic acidosis (MA), which results from intestinal reabsorption of urinary ammonium and promotes bone resorption, has been identified as a key factor in the pathophysiology of bone loss in this setting [8]. Additional risk factors such as advanced age, female sex, type of UD, time since RC, body mass index (BMI), impaired renal function, and metabolic or hormonal alterations (e.g., vitamin D deficiency, hyperparathyroidism, and hypogonadism) may also contribute to reduced bone integrity [8].

Considering the limited existing evidence, this study sought to comprehensively characterize bone health status in male patients with UD by integrating BMD, TBS, and FRAX® and to identify key determinants associated with bone impairment, particularly MA and related clinical factors.

## Materials and methods

### Study design and participants

This cross-sectional and single-center study was conducted between January 2018 and April 2024. Males over 50 years of age who had undergone RC with ileal UD were recruited for evaluation at least 1 year after surgery. Patients with other types of UD, prior diagnosis of osteoporosis, current hemodialysis treatment or history of kidney transplantation, treatments that could affect bone metabolism (antiresorptive or anabolic agents, corticosteroids, androgens, androgen deprivation therapy), or ongoing systemic oncological treatment were not eligible. However, those taking calcium or vitamin D supplements were not excluded.

### Variables

BMD at the lumbar spine (L1–L4), femoral neck, and total hip was expressed in g/cm^2^, T-score and Z-score. These values were classified according to the World Health Organization (WHO) criteria at any of the three analyzed sites: normal (T-score ≥ −1 SD), osteopenia (T-score between −1 and −2.5 SD), and osteoporosis (T-score ≤ −2.5 SD)[12]. Lumbar spine (L1–L4) TBS values were categorized according to cut-off points from a large meta-analysis, which used a gender-independent tertile model [13]. Under this classification, TBS > 1.31 corresponded to the highest tertile (low fracture risk), values between 1.23 and 1.31 to the middle tertile (intermediate risk), and TBS < 1.23 to the lowest tertile (high fracture risk). The presence of vertebral fractures was assessed using the vertebral fracture assessment (VFA). Fracture risk was estimated using the FRAX® tool [14]. In addition, relevant clinical and serum biochemical variables were analyzed.

### Data sources and measurement methods

BMD was measured with a GE-Lunar iDXA densitometer (GE Medical Systems Lunar, Madison, WI, USA). T-scores were calculated using the NHANES III White female reference database (age 20–29 years), in accordance with International Society for Clinical Densitometry (ISCD) recommendations for the diagnostic classification of osteoporosis in both men and women [15]. To enhance epidemiologic comparability and minimize potential misinterpretation, age- and sex-adjusted Z-scores are also reported. TBS was obtained from DXA scans using TBS iNsight software version 3.0.2.0 (Medimaps, France), with TBS T-scores and Z-scores calculated against the manufacturer’s Spanish reference database for adults aged 20–40 years.

All measurements were acquired under a standardized quality control program, following ISCD recommendations and manufacturer guidelines, including daily calibration and periodic phantom scans [15]. Because this was a cross-sectional study, no in vivo precision study was performed, and site-specific least significant change (LSC) values were not calculated. For completeness, we note the ISCD convention that LSC at the 95% confidence level equals 2.77× the precision error [15]. The reported precision of GE-Lunar iDXA is excellent, with coefficients of variation of 1.05% for lumbar spine BMD and 0.99% for total hip BMD.

Presence of vertebral fractures was evaluated from T4 to L5 using VFA. Additional thoracic and lumbar spine X-rays were performed when the primary interpretation was inconclusive. Vertebral fractures were defined according to the Genant semiquantitative method, classifying both deformity type (wedge, biconcave, or crush) and grade based on vertebral height loss: mild (20–25%), moderate (25–40%), or severe (> 40%) [16]. The 10-year risks of major osteoporotic fracture (FRAX-MOF) and hip fracture (FRAX-HIP) were calculated using the Spanish version of the FRAX® tool and subsequently adjusted for femoral neck BMD and TBS [14]. T-scores were entered using White female reference data, as recommended by ISCD/FRAX® guidance [17]. This approach ensures comparability with international epidemiological standards and avoids potential overestimation that could arise from using male reference ranges.

Serum samples were collected between 08:00 and 10:00 h after an overnight fast, in line with published protocols to reduce diurnal and food-related variability. When possible, samples were processed and frozen according to stability recommendations to preserve analyte integrity. Serum C-terminal telopeptide of type 1 collagen (CTX) was measured using an automated electrochemiluminescence immunoassay, specifically the Roche Elecsys β-CrossLaps platform. Samples were processed in a single certified laboratory using a consistent analytical platform. MA was defined as a venous bicarbonate level < 22 mEq/L [18], anemia as hemoglobin < 13 g/dL in men according to WHO criteria, and chronic kidney disease (CKD) as an estimated glomerular filtration rate (eGFR) < 60 mL/min/1.73m^2^.

### Sample size

The sample size was calculated based on an expected prevalence of osteoporosis of 36% in men with UD after RC [7] and 13% in community-dwelling Spanish men [19]. Assuming an alpha error of 0.05 and a beta error below 0.2, the estimated minimum sample size was 58 patients. An adjustment was applied for an estimated dropout rate of 10% by using the ARCSINUS approximation method.

### Statistical analysis

Categorical variables are presented as frequencies and percentages, while continuous variables are summarized as medians with interquartile ranges (IQR). Group comparisons were performed using the chi-square test for categorical variables, the Mann–Whitney *U* test for comparisons of continuous variables between two groups, and the Kruskal–Wallis test for comparisons of continuous variables among more than two groups. All covariates of clinical or biological interest were tested in univariate analyses, and those with a *p* value ≤ 0.1 were included in the multivariable logistic regression models to identify risk factors for osteoporosis and for belonging to the lowest TBS tertile. Associations between variables were assessed with Pearson’s correlation (*r*) when continuous variables were approximately normal and the relationship appeared linear. For ordinal variables or when normality was not met, Spearman’s rank correlation (*ρ*) was used. Statistical analyses were performed using R version 4.3.0 (R Core Team 2021) and RStudio version 2023.03.0 (Build 386). Statistical significance was set at *p* value ≤ 0.05.

### Ethical considerations

This study was approved by the Clinical Research Ethics Committee (CEIm 2019643) and was registered at ClinicalTrials.gov (NCT04153227). All the participants provided written informed consent. The study followed the STROBE guidelines for cross-sectional observational studies [20] and complied with the methodological quality standards for observational research [21].

## Results

### Participant characteristics

A total of 137 patients were initially screened. Of these, 25 did not meet the inclusion criteria and were therefore excluded: 18 were women, 6 had ongoing systemic oncological treatment, 6 had cutaneous ureterostomies, 5 declined to provide informed consent or undergo DXA, 1 had a previous diagnosis of osteoporosis already under treatment before RC, and 1 had a history of kidney transplantation. After applying these exclusion criteria, 112 men with ileal UD were included in the final analysis. Importantly, no participants were withdrawn after study inclusion. The clinical characteristics of the entire study population, including subgroup comparisons across BMD and TBS categories, are summarized in Table 1, while biochemical variables are detailed in Table 2, and bone assessment findings in Table 3; additional data are provided in Supplementary Table S1.

**Table 1:** Clinical characteristics of the total population (*n* = 112) with comparisons according to bone mineral density (BMD) and trabecular bone score (TBS) categories

Clinical variables	Total population (***n*** = 112)	BMD				TBS			
		Normal (***n*** = 27)	Osteopenia (***n*** = 49)	Osteoporosis (***n*** = 36)	***p*** value	TBS > 1.31 (***n*** = 41)	TBS = 1.31–1.23 (***n*** = 23)	TBS < 1.23 (***n*** = 45)	***p*** value
*N* (%)	112 (100%)	27 (24.1%)	49 (43.8%)	36 (32.1%)		41 (36.6%)	23 (20.5%)	45 (40.2%)	
Age, years	71 (64—77)	68 [62.5–74]	68 [63–77]	76 [70.75–80]	** < 0.001**	70 [65–76]	72 [66.5–77]	72 [64–77]	0.959
Type of urinary diversion					0.652				0.071
Bricker	104 (92.9%)	24 (88.9%)	46 (93.9%)	34 (94.4%)		41 (100.0%)	20 (87.0%)	40 (88.9%)	
Neobladder	8 (7.1%)	3 (11.1%)	3 (6.1%)	2 (5.6%)		0 (0%)	3 (13.0%)	5 (11.1%)	
Follow-up time, months	34.78 (14.2–85.6)	45.21 [13.56–83.9]	35.28 [15.45–84.53]	33.75 [14.22–97.78]	0.939	27.62 [14.27–83.74]	46.22 [15.78–90.79]	35.28 [13.91–88.83]	0.913
BMI, kg/m^2^	28.3 (25.6–31.8)	30.84 [27.62–32.66]	29.75 [27.76–33.41]	25.73 [24.32–28]	** < 0.001**	29.72 [26.29–34.01]	26.96 [24.69–28.82]	28.01 [25.59–30.8]	**0.042**
History of bone fracture, *n* (%)	33 (29.5%)	6 (22.2%)	19 (38.8%)	8 (22.2%)	0.163	8 (19.5%)	2 (8.7%)	21 (46.7%)	**0.001**
Family history of osteoporotic fracture, *n* (%)	23 (20.5%)	2 (7.4%)	15 (30.6%)	6 (16.7%)	**0.044**	8 (19.5%)	3 (13.0%)	11 (24.4%)	0.536
Current smoker, *n* (%)	28 (25%)	3 (11.1%)	14 (28.6%)	11 (30.6%)	0.157	5 (12.2%)	8 (34.8%)	14 (31.1%)	0.058
DM, *n* (%)	29 (25.9%)	8 (29.6%)	12 (24.5%)	9 (25.0%)	0.877	8 (19.5%)	5 (21.7%)	13 (28.9%)	0.574
Metabolic acidosis, *n* (%)	16 (14.3%)	4 (14.8%)	8 (16.3%)	4 (11.1%)	0.791	6 (14.6%)	3 (13.0%)	7 (15.6%)	0.962
Vertebral fracture, *n* (%)	11 (9.8%)	1 (3.7%)	2 (4.1%)	8 (22.2%)	**0.009**	1 (2.4%)	3 (13.0%)	7 (15.6%)	0.114

Quantitative variables are expressed as median (IQR) and qualitative variables as number (%). Definitions: Metabolic acidosis defined as venous serum bicarbonate < 22 mEq/L (16); anemia according to WHO criteria (< 13 g/dL in men); CKD defined by eGFR < 60 mL/min/1.73m^2^; BMD according to WHO criteria: normal (T-score ≥ −1 SD); osteopenia (T-score −1 to −2.5 SD); osteoporosis (T-score ≤ −2.5 SD). Note: Statistically significant *p* values are shown in bold

*BMD* bone mineral density, *TBS* trabecular bone score, *BMI* body mass index, *DM* diabetes mellitus, *RC* radical cystectomy

**Table 2:** Biochemical variables in the total population (*n* = 112), with comparisons according to bone mineral density (BMD) and trabecular bone score (TBS) categories

Biochemical variables	Total population (***n*** = 112)	BMD				TBS			
		Normal (***n*** = 27)	Osteopenia (***n*** = 49)	Osteoporosis (***n*** = 36)	***p*** value	TBS > 1.31 (***n*** = 41)	TBS = 1.31–1.23 (***n*** = 23)	TBS < 1.23 (***n*** = 45)	***p*** value
Hemoglobin, g/L	142 (126.75–153)	147 [133–158]	145 [134–157]	136.5 [118.5–147.25]	**0.049**	147 [131–158]	141 [121.5–150.5]	140 [120–149]	0.338
Albumin, g/L	44.6 (41.77–46.73)	45.6 [42.85–47.8]	44.9 [42.4–46.3]	42.95 [40.65–46.5]	0.366	45.3 [41.9–46.8]	44.3 [41.95–46.5]	43.8 [41.7–46.9]	0.195
Calcium, mg/dL	9.6 (9.4–9.9)	9.5 [9.3–9.97]	9.7 [9.5–9.9]	9.6 [9.28–9.72]	0.159	9.65 [9.3–9.9]	9.6 [9.4–9.8]	9.6 [9.4–9.9]	0.969
25 (OH)D, ng/mL	16.6 (12.52–24.10)	14.6 [12.45–21.65]	18.8 [14.2–25]	15.15 [9.18–24.52]	0.452	17.7 [13.1–26.2]	15.8 [11.9–22.2]	16 [12.3–22.5]	0.432
PTH, pg/mL	64 (46.5–84.5)	63 [44–87]	61 [44.5–74.25]	68.5 [56.5–90.25]	0.654	64 [45–86]	61 [43–68.5]	68.5 [51–91]	0.361
ALP, U/L	81 (63.75–101.75)	73 [57.5–86]	74 [61–101]	86 [81–108.75]	**0.003**	79 [59–101]	83 [72.5–104]	78 [61–106]	0.327
CTX, ng/mL	0.377 (0.25–0.55)	0.29 [0.22–0.47]	0.32 [0.22–0.44]	0.48 [0.37–0.67]	**0.027**	0.39 [0.26–0.5]	0.47 [0.36–0.67]	0.32 [0.23–0.5]	0.44
IGF-1, ng/mL	131 (105–173)	130 [114.5–161.75]	133 [101–173.5]	130.5 [110–182]	0.582	132 [110–177]	126 [112–146]	128.5 [95.5–179.75]	0.42
Venous bicarbonate, mEq/L	26.35 (23.90–28.45)	24.9 [23.75–26.9]	26.3 [23.9–28.4]	27.4 [24.6–29.2]	0.134	26.1 [24.3–28.4]	27.4 [26.2–28.85]	25.4 [23.5–28.2]	0.521
Venous base excess, mmol/L	0.2 (−1.80–1.90)	−0.7 [−1.8–0.85]	0.1 [−1.4–2]	1.15 [−1.8–2.55]	0.548	0.2 [−1.4–1.9]	0.6 [−0.05–2.8]	−0.3 [−2.6–1.9]	0.559
Creatinine, mg/dL	1.315 (1.06–1.58)	1.3 [1.05–1.54]	1.27 [1.07–1.57]	1.35 [1.05–1.7]	0.479	1.3 [1.06–1.56]	1.33 [1.02–1.54]	1.34 [1.09–1.82]	0.082
eGFR, mL/min/m^2^	55.5 (42–72.25)	56 [44.5–77]	58 [42–72]	52 [39.25–67.75]	0.305	57 [45–70]	53 [45–75]	52 [39–71]	0.335

Quantitative variables are expressed as median (IQR) and qualitative variables as number (%). Definitions: BMD according to WHO criteria: normal (T-score ≥ −1 SD), osteopenia (T-score −1 to −2.5 SD), osteoporosis (T-score ≤ −2.5 SD). Note: Statistically significant *p* values are shown in bold

*BMD* bone mineral density, *TBS* trabecular bone score, *25(OH)D* 25-hydroxyvitamin D, *PTH* parathyroid hormone, *ALP* alkaline phosphatase, *CTX* C-terminal telopeptide of type 1 collagen, *IGF-1* insulin-like growth factor 1, *eGFR* estimated glomerular filtration rate

**Table 3:** Bone assessment variables in the total population (*n* = 112), with comparisons according to bone mineral density (BMD) and trabecular bone score (TBS) categories

Radiological variables	Total population (***n*** = 112)	BMD				TBS			
		Normal (***n*** = 27)	Osteopenia (***n*** = 49)	Osteoporosis (***n*** = 36)	***p*** value	TBS > 1.31 (***n*** = 41)	TBS = 1.31–1.23 (***n*** = 23)	TBS < 1.23 (***n*** = 45)	***p*** value
Lumbar spine BMD, g/cm^2^	1.13 (0.97–1.27)	1.27 [1.2–1.49]	1.13 [1.03–1.27]	0.93 [0.89–1.05]	** < 0.001**	1.19 [1.06–1.34]	1.05 [0.91–1.25]	1.04 [0.96–1.21]	0.069
Lumbar spine T-score, SD	−0.985 (−2.08–0.21)	0.19 [−0.44–2.02]	−0.99 [−1.77–0.31]	−2.59 [−2.87–1.66]	** < 0.001**	−0.51 [−1.39–0.69]	−1.43 [−2.74–0.04]	−1.57 [−2.22–0.12]	0.07
Femoral neck BMD, g/cm^2^	0.871 (0.73–0.95)	1 [0.97–1.04]	0.88 [0.83–0.9]	0.7 [0.66–0.73]	** < 0.001**	0.88 [0.79–0.98]	0.88 [0.73–0.89]	0.84 [0.71–0.93]	0.149
Femoral neck T-score, SD	−1.5 (−2.57–0.84)	−0.54 [−0.72–0.22]	−1.49 [−1.81–1.27]	−2.83 [−3.17–2.59]	** < 0.001**	−1.26 [−2.13–0.67]	−1.5 [−2.58–1.32]	−1.75 [−2.79–1.26]	0.13
Total hip BMD, g/cm^2^	0.927 (0.8–1.02)	1.08 [1.02–1.13]	0.94 [0.9–0.99]	0.77 [0.73–0.82]	** < 0.001**	0.88 [0.79–0.98]	0.88 [0.73–0.89]	0.84 [0.71–0.93]	0.149
Total hip T-score, SD	−1.24 (−2.2–0.43)	−0.07 [−0.37–0.32]	−1.19 [−1.44–0.6]	−2.45 [−2.79–2.11]	** < 0.001**	−1.26 [−2.13–0.67]	−1.5 [−2.58–1.32]	−1.75 [−2.79–1.26]	0.13
TBS L1-L4	1.274 (1.18–1.35)	1.32 [1.19–1.37]	1.28 [1.18–1.35]	1.25 [1.19–1.31]	0.11	1.37 [1.35–1.44]	1.28 [1.26–1.29]	1.16 [1.13–1.21]	** < 0.001**

Quantitative variables are expressed as median (IQR) and qualitative variables as number (%). Definitions: BMD according to WHO criteria: normal (T-score ≥ −1 SD), osteopenia (T-score −1 to −2.5 SD), osteoporosis (T-score ≤ −2.5 SD). Note: Statistically significant *p* values are shown in bold

*BMD* bone mineral density, *TBS* trabecular bone score, *SD* standard deviation

The median age was 71 years (IQR 64–77), with a postoperative follow-up time of 34.76 months (IQR 14.2–85.6). Heterotopic UD was performed in 92.9% of cases, while the remaining 7.1% received an ileal neobladder. BMI was 28.3 kg/m^2^ (IQR 25.6–31.8). Although 13.4% of the patients were receiving vitamin D supplementation, insufficient (< 30 ng/mL) and deficient (< 20 ng/mL) serum 25-hydroxyvitamin D (25-(OH)D) levels were observed in 89.2% and 58.9% of cases, respectively. High parathyroid hormone (PTH) levels (> 65 pg/mL) were observed in 46.4%. Regarding clinical risk factors included in the FRAX® tool, 29.5% of patients had a history of previous fracture, 20.5% reported a family history of osteoporotic fracture, 25% were current smokers, and 25.9% had a diagnosis of diabetes mellitus.

### Main outcomes: prevalence and distribution of bone deterioration

#### BMD, TBS, vertebral fractures, and fracture risk assessment

The prevalences of osteoporosis and osteopenia were 32.1% and 43.8%, respectively, and only 24.1% had normal BMD values. TBS distribution showed that 40.2% of patients were classified in the lowest tertile, 20.5% in the intermediate tertile, and 36.6% in the highest tertile. TBS estimation was not feasible in three patients due to artifacts associated with high BMI.

**Fig. 1:**
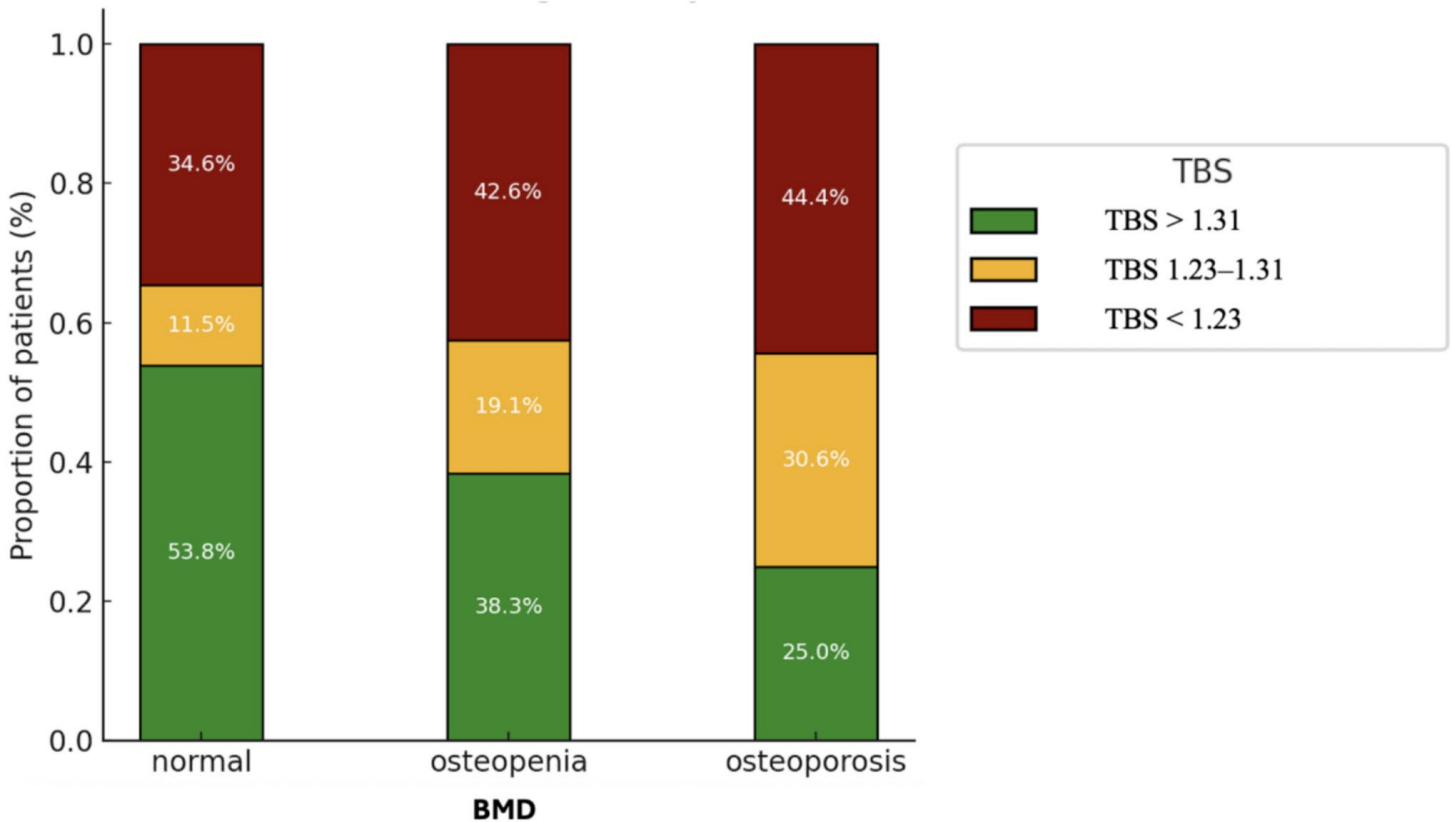
Distribution of TBS categories according to BMD status based on the World Health Organization (WHO) classification. Definitions: BMD according to WHO criteria: normal (T-score ≥ −1 SD); osteopenia (T-score −1 to −2.5 SD); osteoporosis (T-score ≤ −2.5 SD). TBS: highest tertile (> 1.31), middle tertile (1.31–1.23), lowest tertile (< 1.23)

Figure 1 summarizes the distribution of TBS categories across BMD status groups as assessed by DXA. Among patients with osteoporosis, 44.4% were classified in the lowest TBS tertile, 30.6% in the intermediate tertile, and 25% in the highest category. Interestingly, 34.6% of individuals with normal BMD fell into the lowest tertile and 11.5% into the intermediate range, indicating that a considerable proportion of patients with apparently normal BMD may present with lower TBS values. Moreover, only 14 out of 112 patients (12.5%) had normal values for both BMD and TBS. To further explore the relationship between TBS and BMD, correlation analyses were performed. Significant positive correlations were found between TBS and BMD at the lumbar spine (*r* = 0.23, *p* = 0.019), femoral neck (*r* = 0.32, *p* < 0.001), and total hip (*r* = 0.35, *p* < 0.001).

A total of 11 cases with vertebral fractures (9.8%) were identified through VFA, without prior clinical suspicion. Most had a single deformity, while 4/11 (36.4%) presented multiple fractures. Considering all fractures (*n *= 21), wedge morphology predominated (14/21, 66.7%), followed by biconcave (5/21, 23.8%) and crush (2/21, 9.5%). Moderate grades were more frequent (14/21, 66.7%) than severe (7/21, 33.3%). Fractures were mainly thoracic (17/21, 81%) with fewer lumbar events (4/21, 19%). Detailed case-by-case characteristics are provided in Supplementary Table S2.

Median FRAX-MOF and FRAX-HIP were 3.9% (IQR 2.7–6.05) and 1.35% (IQR 0.7–2.78), respectively (Table 4). When adjusted for BMD, fracture risk estimates increased substantially, particularly in patients with osteoporosis, where FRAX-MOF and FRAX-HIP reached median values of 9.55% (IQR 6–14) and 5.8% (IQR 3.48–9), respectively. Both values exceeded the treatment thresholds defined by the Spanish Society for Rheumatology (SER) (> 7.5% for major fracture and > 3% for hip fracture)[22]. After TBS adjustment, estimated fracture risks increased further. In patients with osteoporosis, FRAX-MOF reached a median of 9.95% and FRAX-HIP 5.85%, reinforcing the high-risk profile of this subgroup. Markedly, in individuals with the lowest TBS values, the median adjusted risk also exceeded SER thresholds (FRAX-MOF 8.7% and FRAX-HIP 3%), despite not all of them being classified as osteoporotic by BMD. In contrast, patients outside the lowest TBS tertile did not meet treatment thresholds unless osteoporosis was also present.

**Table 4:** 10-year probability of fracture according to FRAX® in the total population (*n* = 112), with comparisons according to bone mineral density (BMD) and trabecular bone score (TBS) categories

Risk of fracture	Total population (***n*** = 112)	BMD				TBS			
		Normal (***n*** = 27)	Osteopenia (***n*** = 49)	Osteoporosis (***n*** = 36)	***p*** value	TBS > 1.31 (***n*** = 41)	TBS = 1.31–1.23 (***n*** = 23)	TBS < 1.23 (***n*** = 45)	***p*** value
FRAX-MOF without BMD	3.9 (2.7–6.05)	2.8 [2.4–3.9]	3.8 [2.5–6.3]	5.55 [4.05–7.6]	**0.011**	3.4 [2.7–4.8]	4.1 [2.65–5.75]	4.8 [3.5–7.4]	0.153
FRAX-MOF with BMD	5.3 (2.98–7.82)	2.6 [2.15–3.1]	5.1 [3.4–6.6]	9.55 [6–14]	** < 0.001**	3.8 [2.8–6.6]	5.4 [3.3–7.75]	6.4 [4.1–12]	**0.011**
FRAX-MOF adjusted with TBS	5.8 (3.4–9.5)	2.95 [2.5–3.4]	5.7 [3.95–8.55]	9.95 [6.65–16]	** < 0.001**	4 [2.8–6.3]	5.8 [3.85–8.35]	8.7 [5.4–14]	** < 0.001**
FRAX-HIP without BMD	1.35 (0.7–2.78)	1 [0.4–1.55]	1.2 [0.5–2.4]	2.7 [1.78–4.35]	**0.019**	1.1 [0.5–2.2]	1.5 [0.75–2.8]	1.7 [0.9–3.5]	0.431
FRAX-HIP with BMD	1.8 (0.9–4.55)	0.7 [0.2–0.95]	1.6 [1.1–2.6]	5.8 [3.48–9]	** < 0.001**	1.2 [0.7–3.6]	2.2 [1.15–4.45]	2.6 [1–6.9]	**0.022**
FRAX-HIP adjusted with TBS	2 (1–5.2)	0.8 [0.3–1.08]	1.8 [1.1–3.2]	5.85 [3.5–8.6]	** < 0.001**	1 [0.7–3.2]	2.5 [1.25–4.55]	3 [1.5–7.2]	**0.005**

Quantitative variables are expressed as median (IQR). Definitions: BMD according to WHO criteria: normal (T-score ≥ −1 SD), osteopenia (T-score −1 to −2.5 SD), osteoporosis (T-score ≤ −2.5 SD). Note: Statistically significant *p* values are shown in bold

*BMD* bone mineral density, *TBS* trabecular bone score, *FRAX* Fracture Risk Assessment Tool, *FRAX-MOF* the 10-year risks of major osteoporotic fracture, *FRAX-HIP* the 10-year risks of hip fracture

### Subgroup comparisons

#### Osteoporosis group

Patients with osteoporosis were significantly older, had a lower BMI, and had a higher frequency of vertebral fractures compared to the other groups (Table 1). While no significant differences were observed in other demographic characteristics, this group showed lower hemoglobin levels on biochemical analysis (Tables 1 and 2). However, this was not accompanied by a higher prevalence of anemia. In contrast, CTX levels were significantly higher in the osteoporosis group. The FRAX-MOF and FRAX-HIP scores, with and without adjustment for BMD and TBS, were also significantly higher (Table 4).

#### Lowest TBS tertile group

Patients classified in the lowest TBS tertile (TBS < 1.23) had lower BMI and a greater frequency of prior fractures compared with those in the intermediate or highest tertiles (Table 1). No significant differences were observed in other radiological or biochemical parameters (Tables 2 and 3), nor in the FRAX® estimates without BMD adjustment (Table 4).

#### Association with MA

MA was observed in 16 patients (14.3%). These individuals exhibited significantly lower levels of hemoglobin, albumin, calcium, 25-hydroxyvitamin D, and testosterone, as well as higher concentrations of PTH, phosphorus, CTX, creatinine, and eGFR than non-acidotic patients. However, no significant differences were observed in BMD, TBS, VFA, or FRAX® values or in demographic characteristics between patients with and without MA (see Supplementary Table S3). Additionally, no significant correlations were found between venous bicarbonate levels and PTH (*ρ* = −0.04, *p* = 0.654), BMD values at the spine (*ρ* = −0.17, *p* = 0.082), femoral neck (*ρ* = −0.17, *p* = 0.07) or total hip (*ρ* = −0.17, *p* = 0.07), and TBS (*ρ* = 0.07, *p* = 0.439).

### Univariate and multivariate analysis

#### Factors associated with osteoporosis

In univariate analysis, age (per year, OR = 1.12, 95% CI 1.05–1.20; *p* < 0.001), BMI (per kg/m^2^, OR = 0.75, 95% CI 0.66–0.86; *p* < 0.001), and hemoglobin level (per g/L, OR = 0.98, 95% CI 0.96–1.00; *p* = 0.018) were associated with osteoporosis. After adjustment, only age (per year, OR = 1.11, 95% CI 1.02–1.20; *p* = 0.012) and BMI (per kg/m^2^, OR = 0.73, 95% CI 0.62–0.86; *p* < 0.001) remained independent predictors (Table 5).

**Table 5:** Univariate and multivariate analyses of factors associated with osteoporosis (A) and with the lowest tertile of trabecular bone score (TBS < 1.230) (B)

		Univariate			Multivariate	
Variable	OR	95% CI	***p*** value	OR	95% CI	***p*** value
A						
Age (per years)	1.12	1.05–1.20	** < 0.001**	1.11	1.02–1.20	** < 0.001**
BMI (per kg/m^2^)	0.75	0.66–0.86	** < 0.001**	0.73	0.62–0.86	** < 0.001**
Hemoglobin (per g/L)	0.98	0.96–1.00	**0.018**	1.01	0.98–1.04	0.645
Venous bicarbonate (per mEq/L)	1.13	1–1.29	0.057	0.97	0.78–1.21	0.794
B						
BMI (per kg/m^2^)	0.92	0.85–1	0.064	0.91	0.83–0.99	**0.041**
Creatinine (per mg/dL)	1.96	1.04–3.69	**0.038**	2.11	1.08–4.11	**0.028**
History of bone fracture (yes vs. no)	3.94	1.69–9.58	**0.001**	5.23	2.08–13.13	** < 0.001**

Osteoporosis was defined as a T-score ≤  − 2.5 SD at any measured site (lumbar spine, femoral neck, or total hip), according to WHO diagnostic criteria. Note: Only variables with *p* < 0.1 in univariate analysis were included in the multivariate model

*TBS* trabecular bone score, *BMI* body mass index, *OR* odds ratio, *CI* confidence interval

#### Factors associated with TBS in the lowest tertile

Serum creatinine (per mg/dL, OR = 1.96, 95% CI 1.04–3.69; *p* = 0.038), BMI (per kg/m^2^, OR = 0.91, 95% CI 0.83–0.99; *p* = 0.041), and history of prior fracture (OR = 3.94, 95% CI 1.69–9.58; *p* < 0.001) were associated with the lowest TBS tertile in the univariate analysis. All three remained independently associated in multivariate model: serum creatinine (per mg/dL, OR = 2.11, 95% CI 1.08–4.11; *p* = 0.028), BMI (per kg/m^2^, OR = 0.91, 95% CI 0.83–0.99; *p* = 0.041), and prior fracture (OR = 5.23, 95% CI 2.08–13.13; *p* < 0.001) (Table 5).

## Discussion

In our cohort of 112 men with ileal UD after RC, osteoporosis was present in 32.1% of patients, and 40.2% were classified within the lowest TBS tertile, a distribution consistent with a skeletal profile associated with increased fracture risk. To our knowledge, this represents one of the most comprehensive assessments of bone health in patients with UD, integrating BMD, TBS, and fracture risk estimation using the FRAX® tool. Notably, only 12.5% of patients exhibited both preserved BMD and higher TBS values, suggesting that a substantial proportion of individuals with UD may exhibit skeletal alterations relevant to fracture risk.

This study focused exclusively on male patients to avoid the confounding effects of menopause-induced skeletal changes, allowing a more homogeneous assessment of UD-related effects. This methodological choice is further supported by our previous study on MA, in which none of the few female participants met diagnostic criteria [23]. Restricting the sample to men enhances internal consistency and strengthens the reliability of our analysis regarding MA and its impact on bone health.

Although bone health has been examined in patients with UD, few studies have specifically assessed BMD, and available data on osteoporosis remain limited. The prevalence of osteoporosis in our cohort was like the 36% reported by Giannini et al. [7] in 25 men with orthotopic ileal neobladders, based on T-score < −2.5 SD at the femoral neck. Interestingly, their reported rate at the lumbar spine was 32%, also closely matching our findings. However, the generalizability of their results is limited due to the small number of participants. In contrast, two other studies with also limited sample sizes reported no cases of osteoporosis among UD patients [5, 6]. These discrepancies highlight the need for standardized and comprehensive approaches to bone health evaluation. Moreover, although we did not include a control group of healthy men, indirect comparisons with other reports suggest that the proportion of affected individuals in our study far exceeds the 13% reported in healthy Spanish men [19] and nearly threefold higher than in a demographically comparable group [24].

TBS is a non-invasive imaging tool derived from gray-level variations in lumbar spine DXA images. It provides a texture-based parameter that complements BMD in the assessment of skeletal status [19]. Previous studies have reported associations between TBS values and fragility fractures, including in individuals without osteoporosis by BMD criteria [9, 25]. To date, however, no study has specifically evaluated TBS in patients with UD. Although TBS and BMD assess different aspects of bone health, modest to moderate correlations were observed in our cohort between both parameters at all measured sites, suggesting a consistent trend whereby lower TBS values are generally associated with lower BMD**.** The mean TBS was 1.269 ± 0.14 (median 1.274), closely matching values reported in large population datasets [13], with a mean TBS Z-score of 0.0, indicating no significant deviation from expected values for age and sex. Compared to the CAMARGO study, which evaluated over 1000 Spanish men over 50 years of age and reported a mean TBS of 1.345, our patients with UD showed markedly lower TBS values [26], despite having a lower reported rate of vertebral fractures (21.3% vs. 9.8%) [19]. Differences in mean age between cohorts (65 vs. 70.6 years) may partly explain this apparent discrepancy. Notably, 40.2% of participants were classified in the lowest tertile and 36.6% in the highest, while 46.2% of those with non-osteoporotic BMD also had low TBS values. These proportions should be interpreted considering the methodological limitations of tertile-based cut-offs, which may overstate differences [9, 13]. These findings support the potential complementary role of TBS alongside BMD in skeletal risk stratification.

The FRAX® tool was developed to estimate the 10-year probability of major osteoporotic and hip fractures [10]. Although BMD is a well-established predictor of fracture risk, FRAX® enhances risk estimation by incorporating additional clinical variables [27]. In our patients, BMD reduction was significantly associated with an increased fracture risk, even when the algorithm was not adjusted for BMD or TBS. This finding is consistent with previous research showing that low BMD is a strong independent predictor of fragility fractures [28, 29]. However, when unadjusted scores were analyzed, no clear differences emerged across TBS tertiles. Integrating BMD into FRAX® improves its discriminative power over clinical risk factors alone [30], and further adjustment with TBS enhances predictive accuracy [13], supporting its combined use for optimal fracture risk evaluation and clinical decision-making. The addition of TBS to FRAX may enhance the detection of high-risk individuals, particularly those who do not meet osteoporosis criteria based on BMD alone. This is especially relevant in cohorts where reduced TBS values are observed even in the absence of osteoporosis by BMD, as fracture risk estimates in these individuals may still exceed treatment thresholds when TBS is incorporated. Despite growing evidence of increased fracture risk in patients with UD, the use of FRAX® in this setting has neither been formally evaluated, unlike in prostate cancer, where routine fracture risk assessment is advised, particularly before initiating androgen deprivation therapy [22, 31].

Patients with bladder cancer frequently exhibit multiple risk factors for fracture, many of which are already included in the FRAX® tool [10, 11]. Hormonal and molecular imbalances may contribute to alterations in skeletal properties. Although vitamin D deficiency was highly prevalent among study participants and is commonly observed in patients with UD [32], serum 25-(OH)D levels did not show a significant association with osteoporosis in our univariate analysis. Nevertheless, given the very low median 25-(OH)D levels observed, it remains possible that vitamin D insufficiency may adversely influence skeletal integrity through mechanisms such as osteomalacia. Importantly, however, none of the patients exhibited biochemical abnormalities typically suggestive of overt osteomalacia, such as hypocalcemia or isolated elevations of alkaline phosphatase. Similarly, secondary hyperparathyroidism had no substantial negative effect, which is consistent with previous findings [8]. Other key regulators of bone physiology, such as testosterone, estradiol, and insulin-like growth factor 1 (IGF-1), did not demonstrate a determining influence on bone parameters. These findings indicate that the direct influence of these biochemical factors may be limited in patients with UD, suggesting that multifactorial or alternative mechanisms deserve closer examination. Further investigation is needed to clarify these potential contributors.

MA has been identified as a risk factor for both osteoporosis and bone fractures in patients with UD [3, 4, 8, 33], with significant correlations reported between MA and BMD values or biochemical markers of bone metabolism [7, 8, 34–38]. Chronic MA promotes calcium and phosphate mobilization from bone as a compensatory mechanism to buffer excess protons, leading to increased osteoclastic activity and reduced bone formation. This effect may be further intensified by renal insufficiency [1, 23], and it could negatively impact both BMD and TBS [3, 4, 7]. However, in our study, although patients with MA showed greater hyperparathyroidism and higher CTX levels, they did not exhibit an increased prevalence of osteoporosis, lowest TBS values, or vertebral fractures than non-acidotic individuals. Neither MA nor venous bicarbonate levels emerged as significant risk factors for osteoporosis or TBS values within the lowest tertile. The lack of significant correlations between MA and bone parameters in our analysis contrasts with previous reports [7, 34–38]. These discrepancies may be partly explained by methodological factors, since acid–base evaluation relied on venous rather than arterial parameters, which are more susceptible to physiological and technical variability [39]. Moreover, interpreting CTX levels in advanced renal impairment is challenging. Recent consensus statements highlight that serum CTX may be elevated in CKD due to decreased renal clearance [40]. Given that all patients with MA in our study had substantial renal dysfunction, biochemical abnormalities such as elevated PTH and phosphorus may be influenced, at least in part, by CKD-related mineral disturbances rather than acidosis alone, and the association between MA and higher CTX levels should be interpreted with caution. Collectively, these findings challenge the role of MA as the primary driver of bone loss in this context, suggesting a multifactorial etiology.

Aging is one of the main contributors to bone loss [22]. RC is predominantly performed in older adults, a group already vulnerable to bone loss and fracture. Previous studies have reported a significant impact of age on bone health in patients with UD [7, 41, 42], with fracture risk notably increasing in individuals over 75 years [3, 4]. In addition, age-related frailty in oncologic patients may favor the development of sarcopenia, falls, and fractures, thereby contributing to increased overall mortality rates. We found that age correlated significantly with femoral neck BMD and total hip BMD, but not with lumbar spine BMD or TBS (see Supplementary Fig. S1). This could be partially explained by the presence of degenerative changes in the spine of older patients, potentially affecting BMD measurements at this site. Moreover, advanced age emerged as an independent risk factor for osteoporosis in the multivariable analysis but was not independently associated with the lowest TBS category.

BMI is a relevant determinant of bone health, with few documented associations with BMD in patients with UD [8]. Poulsen et al.[42] found a significant linear correlation between weight, height, and total body BMD in patients with neobladders and healthy controls. In our study, BMI showed significant correlations with both BMD and TBS (see Supplementary Fig. S2) and emerged as an independent protective factor against osteoporosis and lower TBS values. These results differ from those reported by Schousboe et al.[43], who found an inverse relationship between BMI and TBS in a large cohort of older men, possibly due to soft tissue artifacts affecting TBS measurements.

Fracture history itself is a well-established predictor of future fracture risk, as demonstrated in a large meta-analysis, which forms the basis for its inclusion as a key variable in the FRAX® algorithm [44]. In our study, previous fracture history emerged as an independent risk factor for being classified in the lowest TBS tertile, but not for osteoporosis, underlining its importance in identifying skeletal fragility beyond what is captured by BMD alone. In a population-based analysis from the National Health and Nutrition Examination Survey (NHANES), older Caucasian men with prior fractures had significantly lower TBS values, even after adjusting for age, BMI, and BMD [45]. Similarly, a previous comprehensive review concluded that TBS is independently associated with both prior and incident fractures [46], reinforcing its clinical value beyond BMD-based assessment.

Impaired renal function adversely affects skeletal integrity and is associated with an increased risk of fractures [47]. Although CKD is a known contributor to bone loss and is associated with both MA and vitamin D deficiency [23], its overall impact on BMD and TBS within our patients was modest (see Supplementary Fig. S3). Nonetheless, higher serum creatinine levels remained independently linked to lower TBS values. Likewise, despite robust evidence linking diabetes mellitus to altered skeletal properties and increased fracture risk [48], no significant association with BMD or TBS was observed in the present analysis.

Finally, low hemoglobin levels have been linked to reduced BMD and increased fracture risk [49]. In addition to their skeletal implications, they have also been associated with poorer oncological outcomes following RC [50]. While we did not identify hemoglobin as an independent risk factor for osteoporosis or lower TBS values, it was correlated significantly with femoral neck BMD, total hip BMD, and TBS, but not with lumbar spine BMD (see Supplementary Fig. S4). This observation suggests that hemoglobin may serve as an accessible marker associated with skeletal fragility in this clinical setting.

## Strengths and limitations

Our study has several strengths. To our knowledge, this is the largest published study assessing BMD, TBS, fractures, and FRAX® in patients with UD after RC. The exclusive inclusion of male patients reinforces the internal validity by eliminating the confounding effect of menopause on bone metabolism as an additional factor of osteoporosis.

Nevertheless, our study has some limitations. First, the absence of a healthy matched control group represents the main limitation of the study. This precludes direct comparison with non-UD individuals and limits our ability to discern the specific contribution of ileal UD to skeletal impairment; however, our findings were contextualized using published population-based data, which consistently show lower prevalence rates of osteoporosis, supporting the clinical relevance of our results. Second, the cross-sectional design precludes temporal or causal inference. Third, the male-only cohort prevents extrapolation to female patients with UD. Fourth, the use of FRAX® thresholds calibrated to the Spanish population may not fully capture fracture risk in other geographical or ethnic settings. Fifth, the evaluation of acid–base status was based on venous parameters, which may be influenced by additional factors and thus limit their interpretation in relation to bone outcomes. Moreover, the interpretation of CTX values in patients with advanced renal impairment is complex, as reduced eGFR elevates serum CTX. Consequently, any potential association between MA and CTX levels in our study is likely confounded by renal impairment and cannot be reliably interpreted. Finally, potential survivor bias cannot be excluded, as patients with more aggressive cancer or severe comorbidities may have been underrepresented.

Despite these limitations, our study provides novel insights by simultaneously evaluating BMD, TBS, and FRAX® in individuals at high but under-recognized risk for bone fragility and supports the implementation of routine skeletal assessment in patients undergoing UD. Future prospective studies should validate these findings in larger and more heterogenic cohorts and should explore the impact of early interventions on fracture incidence in these patients. A better understanding of the multifactorial nature of increased fracture risk could open new preventive and therapeutic avenues.

## Conclusions

Our findings indicate that men with UD after RC commonly met diagnostic criteria for osteoporosis and showed a meaningful proportion of TBS values within the lowest tertile, consistent with increased skeletal fragility and fracture risk. Although MA correlated with adverse biochemical profiles, it was not independently associated with adverse bone outcomes. In contrast, clinical factors such as age, BMI, serum creatinine, and prior fracture history emerged as significant predictors of osteoporosis and low TBS values. These findings support consideration of structured bone health assessment and management in patients with UD.

## Supplementary Information

Below is the link to the electronic supplementary material.

ESM 1 (DOCX 379 KB)

ESM 2 (DOCX 354 KB) 

ESM 3 (DOCX 813 KB)

ESM 4 (DOCX 704 KB)

ESM 5 (DOCX 24.4 KB)

ESM 6 (DOCX 26.6 KB)

ESM 7 (DOCX 18.6 KB)

## Data Availability

The datasets generated and/or analysed during the current study are available from the corresponding author on reasonable request.
