# Supplementary material for: Fracture risk assessment in patients with ileal urinary diversion after radical cystectomy: a comprehensive evaluation integrating bone mineral density, trabecular bone score, and FRAX®
Source: Arch Osteoporos. 2026 Mar 11;21(1):50. doi: 10.1007/s11657-026-01685-x (PMC12979281; doi:10.1007/s11657-026-01685-x)

**Fig. S2** Correlations between body mass index (BMI) and bone mineral density (BMD) of the lumbar spine (**A**), femoral neck (**B**), total hip (**C**), and trabecular bone score (TBS) (**D**).


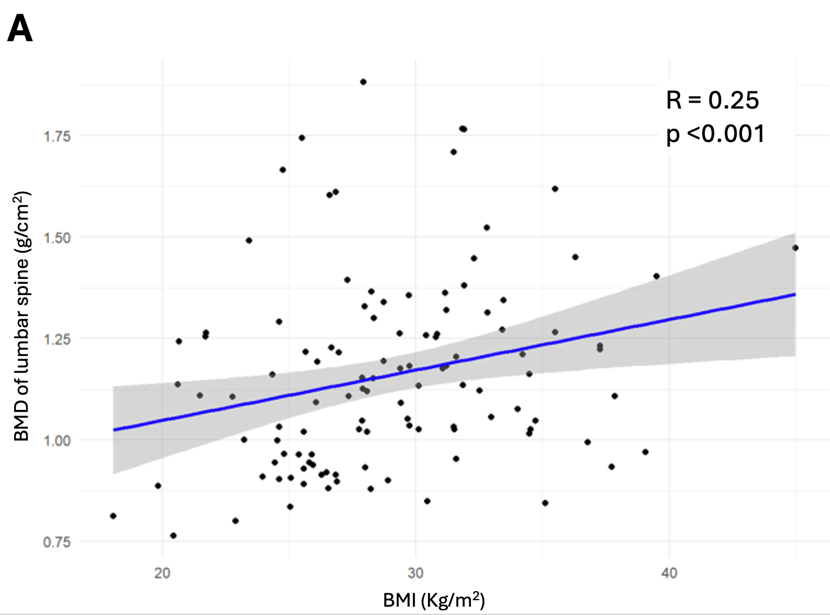


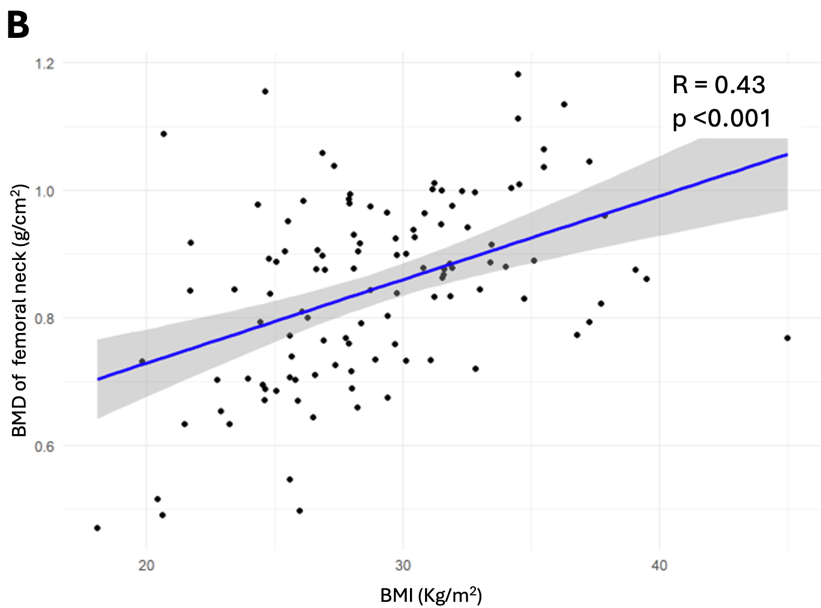


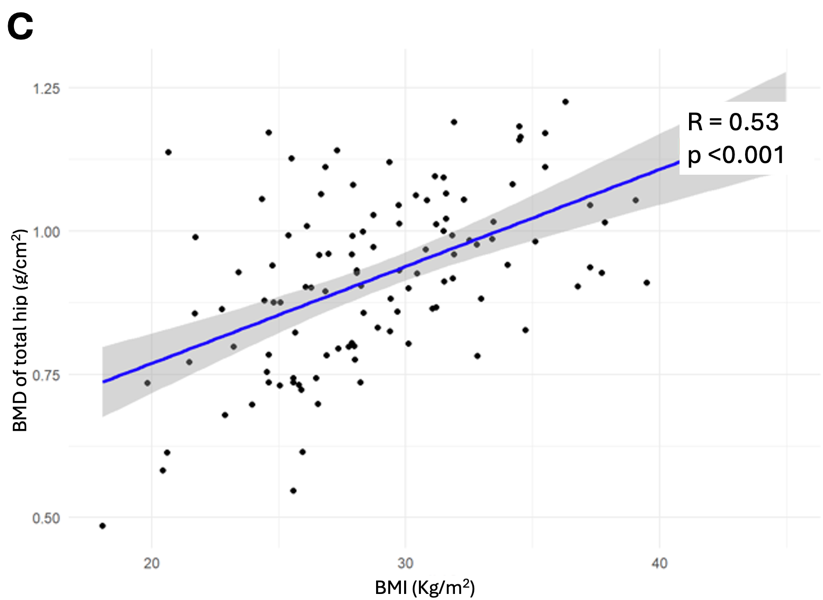


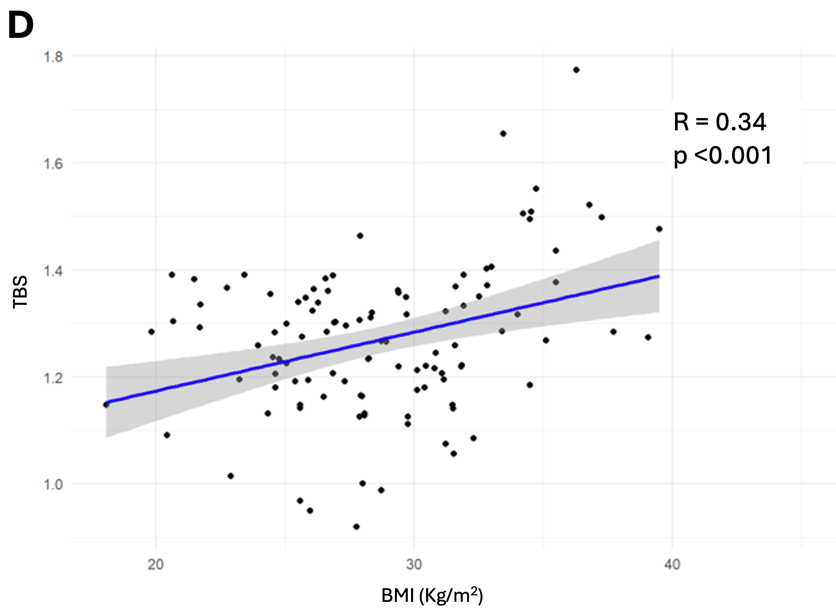

Supplement: Supplementary file 2 — (DOCX 354 KB) [file 11657_2026_1685_MOESM2_ESM.docx]
