# Supplementary material for: Fracture risk assessment in patients with ileal urinary diversion after radical cystectomy: a comprehensive evaluation integrating bone mineral density, trabecular bone score, and FRAX®
Source: Arch Osteoporos. 2026 Mar 11;21(1):50. doi: 10.1007/s11657-026-01685-x (PMC12979281; doi:10.1007/s11657-026-01685-x)

**Fig. S3** Correlations between creatinine and parathyroid hormone (PTH) (**A**), venous serum bicarbonate (**B**), bone mineral density (BMD) of the lumbar spine (**C**), femoral neck (**D**), total hip (**E**), and trabecular bone score (TBS) (**F**).


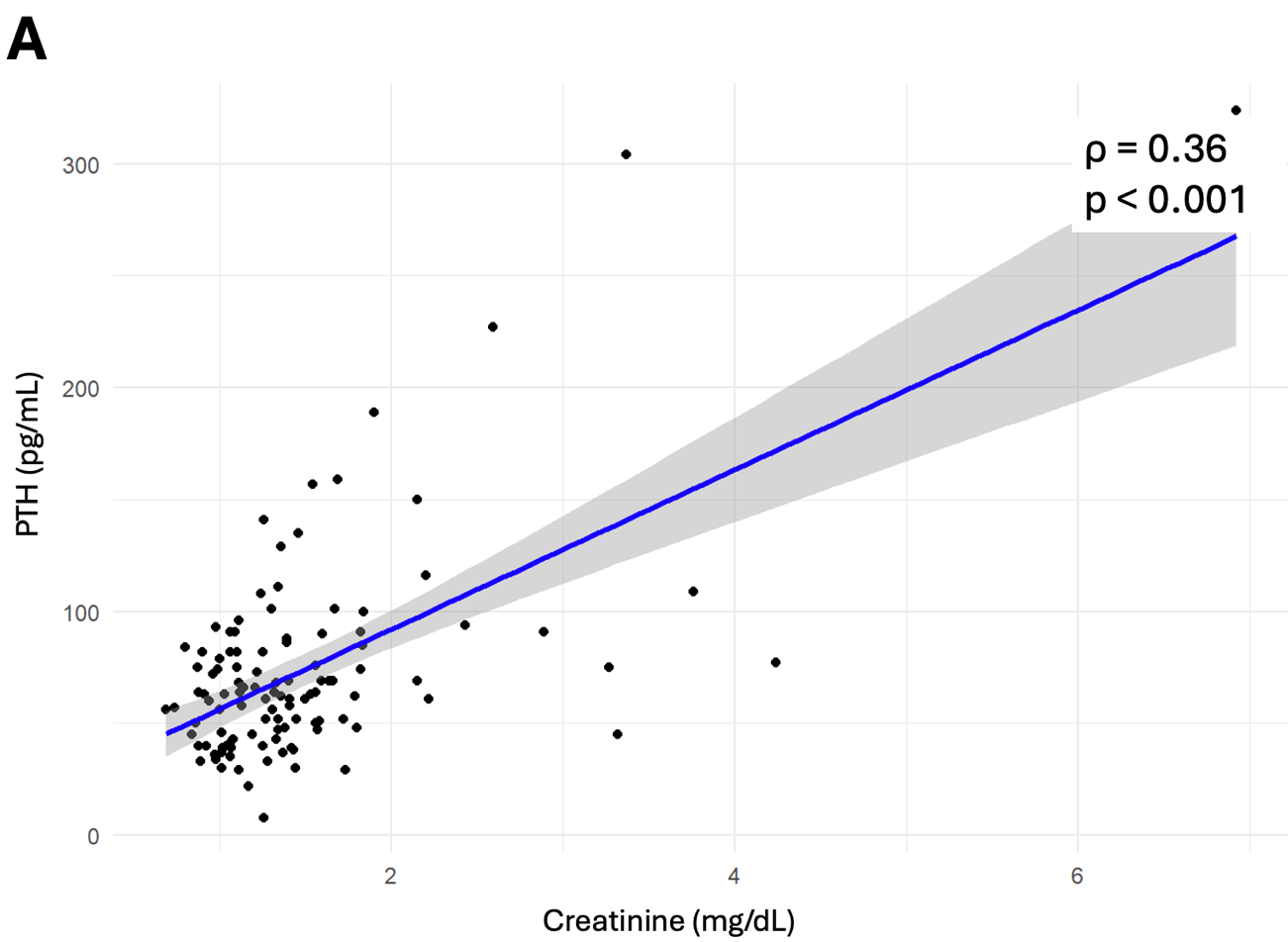


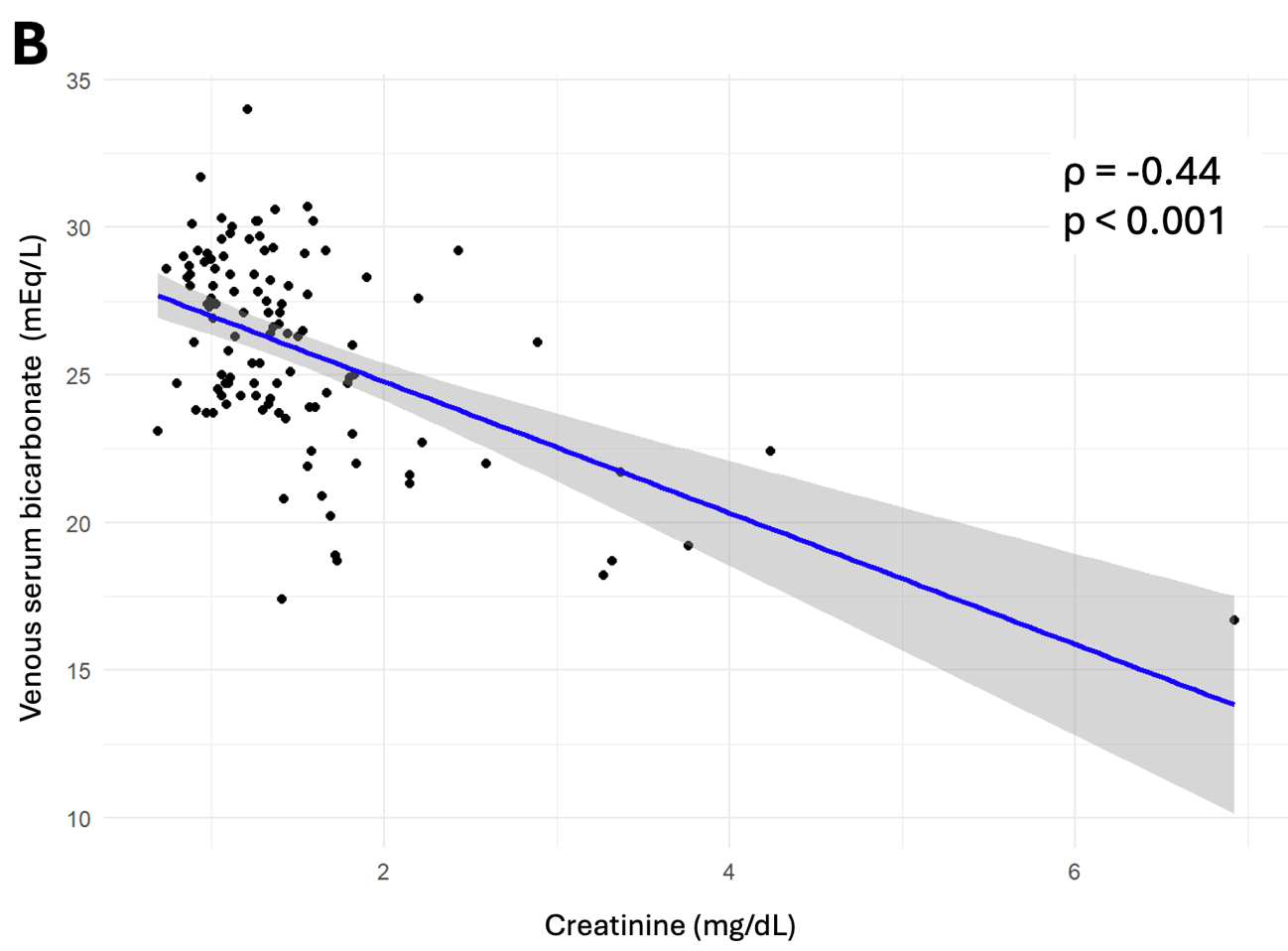


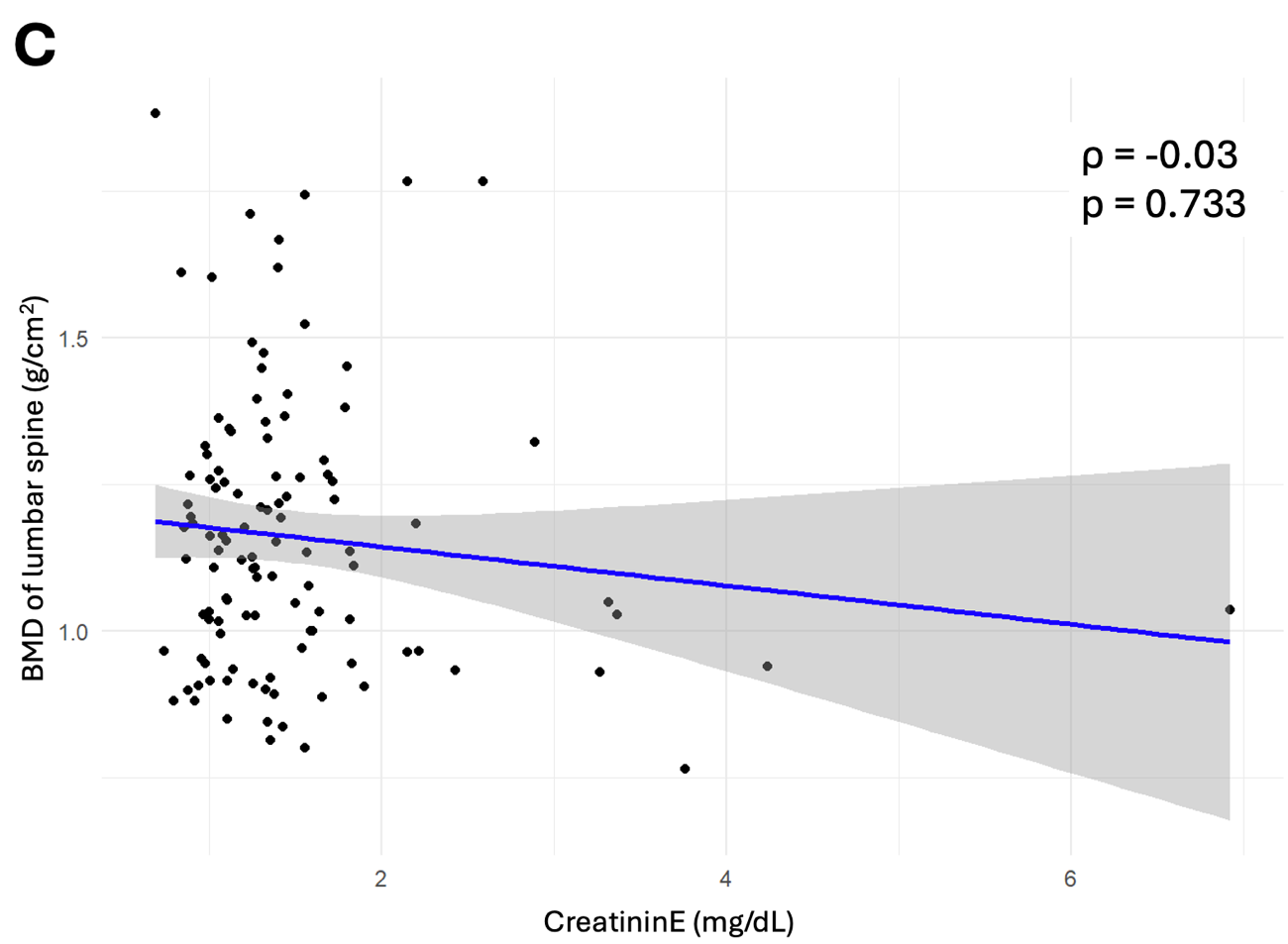


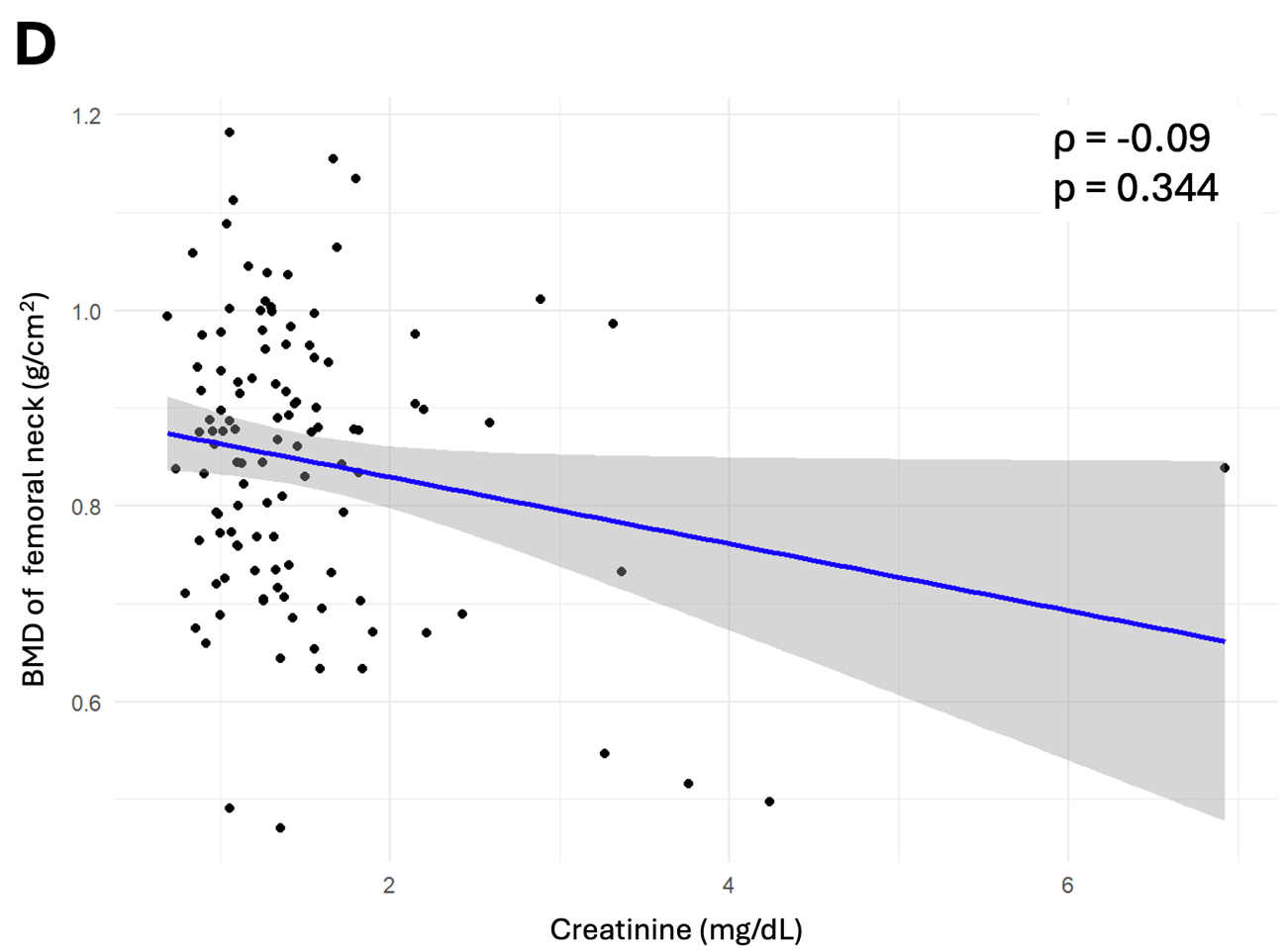


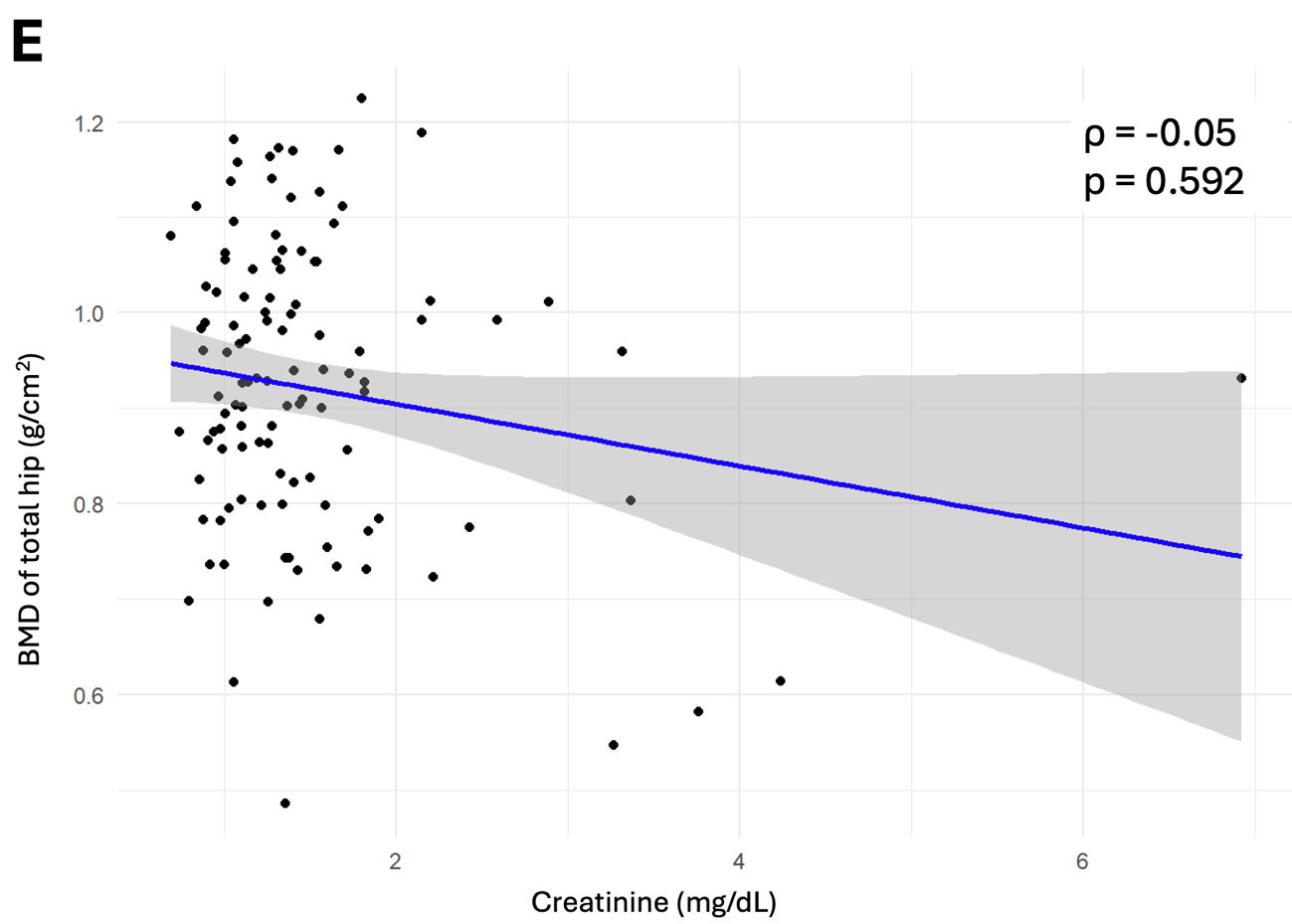


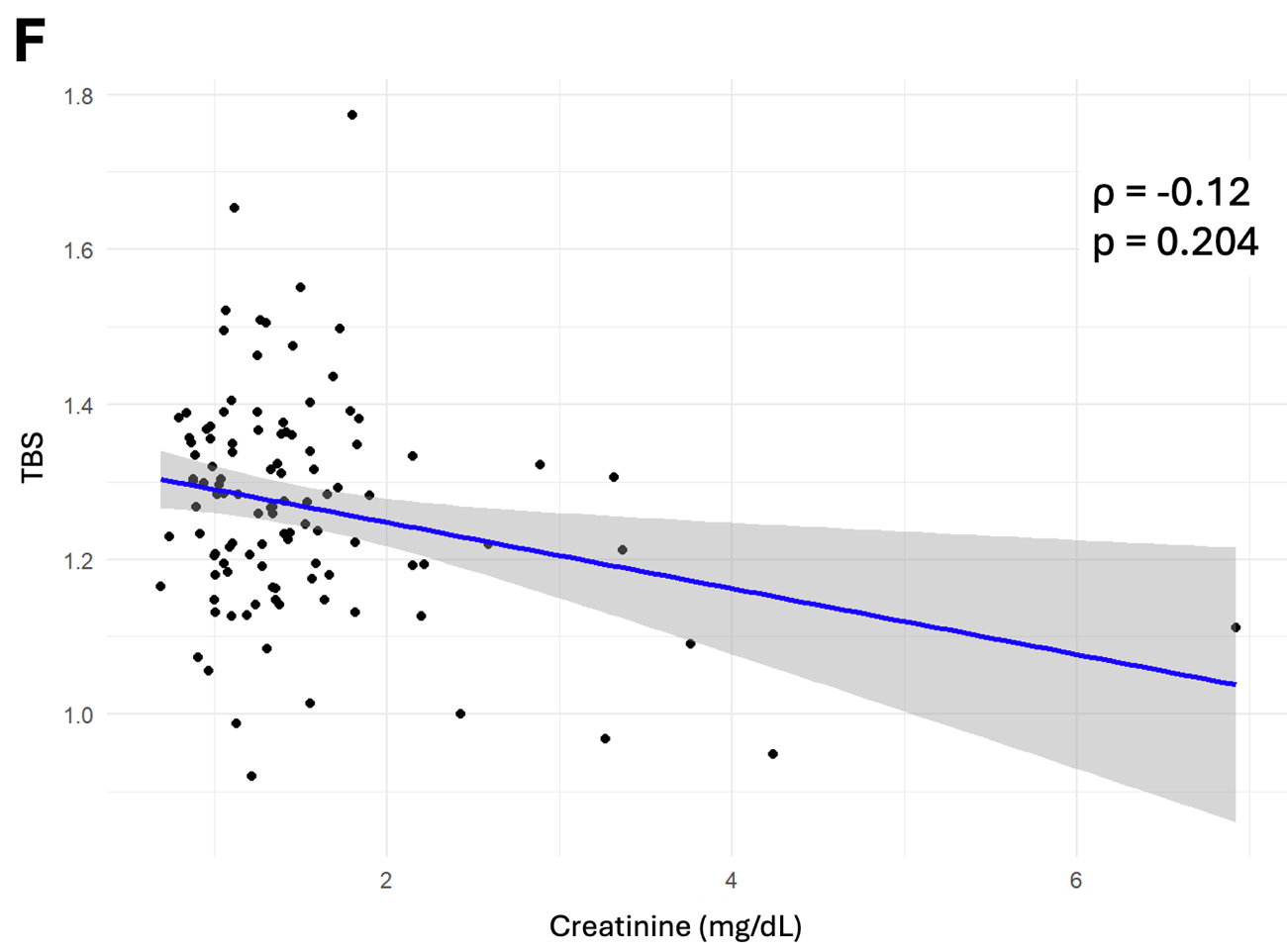

Supplement: Supplementary file 3 — (DOCX 813 KB) [file 11657_2026_1685_MOESM3_ESM.docx]
