# Supplementary material for: Fracture risk assessment in patients with ileal urinary diversion after radical cystectomy: a comprehensive evaluation integrating bone mineral density, trabecular bone score, and FRAX®
Source: Arch Osteoporos. 2026 Mar 11;21(1):50. doi: 10.1007/s11657-026-01685-x (PMC12979281; doi:10.1007/s11657-026-01685-x)
Supplement: Supplementary file 5 — (DOCX 24.4 KB) [file 11657_2026_1685_MOESM5_ESM.docx]

**Supplementary Table S1.** Comparison of clinical, biochemical, and radiological variables in the total population (n=112), with comparisons according to bone mineral density (BMD) and trabecular bone score (TBS) categories.

|  |  |  | **BMD** |  |  |  | **TBS** |  |  |
| --- | --- | --- | --- | --- | --- | --- | --- | --- | --- |
| **Clinical variables** | **Total population**  **(n=112)** | **Normal**  **(n=27)** | **Osteopenia**  **(n=49)** | **Osteoporosis**  **(n=36)** | **p-value** | **TBS > 1.31**  **(n=41)** | **TBS = 1.31-1.23 (n=23)** | **TBS < 1.23**  **(n=45)** | **p-value** |
| **Dietary calcium intake, mg** | 650 (500-800) | 700 [450-800] | 650 [487.5-785] | 700 [500-850] | 0.479 | 650 [450-800] | 750 [550-820] | 675 [437.5-800] | 0.227 |
| **Glucocorticoid therapy, n (%)** | 3 (2.7 %) | 1 (3.7 %) | 1 (2.0 %) | 1 (2.8 %) | 0.911 | 2 (4.9 %) | 1 (4.3 %) | 0 (0%) | 0.335 |
| **Alcohol >3 U/d, n (%)** | 10 (8.9 %) | 4 (14.8 %) | 2 (4.1 %) | 4 (11.1 %) | 0.249 | 3 (7.3 %) | 2 (8.7 %) | 5 (11.1 %) | 0.828 |
| **Rheumatoid arthritis, n (%)** | 1 (0.9 %) | 1 (3.7 %) | 0 (0%) | 0 (0%) | 0.204 | 1 (2.4 %) | 0 (0%) | 0 (0%) | 0.433 |
| **Secondary osteoporosis, n (%)** | 4 (3.6 %) | 1 (3.7 %) | 2 (4.1 %) | 1 (2.8 %) | 0.949 | 2 (4.9 %) | 0 (0%) | 2 (4.4 %) | 0.571 |
| **Anemia, n (%)** | 16 (14.3 %) | 7 (25.9 %) | 10 (20.4 %) | 13 (36.1 %) | 0.269 | 10 (24.4 %) | 7 (30.4 %) | 13 (28.9 %) | 0.843 |
| **CKD prior to RC, n (%)** | 15 (13.4 %) | 4 (14.8 %) | 3 (6.1 %) | 8 (22.2 %) | 0.095 | 5 (12.2 %) | 1 (4.3 %) | 8 (17.8 %) | 0.29 |
| **CKD after RC, n (%)** | 61 (54.5 %) | 17 (63 %) | 24 (49 %) | 20 (55.6 %) | 0.497 | 23 (56.1 %) | 11 (47.8 %) | 25 (55.6 %) | 0.791 |
| **Metabolic acidosis, n (%)** | 16 (14.3 %) | 4 (14.8 %) | 8 (16.3 %) | 4 (11.1 %) | 0.791 | 6 (14.6 %) | 3 (13.0 %) | 7 (15.6 %) | 0.962 |
| **Bicarbonate therapy, n (%)** | 4 (3.6 %) | 1 (3.7 %) | 3 (6.1 %) | 0 (0 %) | 0.323 | 1 (2.4 %) | 1 (4.3 %) | 2 (4.4 %) | 0.868 |
| **Fracture after RC n (%)** | 3 (2.7 %) | 1 (3.7 %) | 0 (0%) | 0 (0%) | 0.426 | 0 (0%) | 0 (0%) | 3 (6.7 %) | 0.356 |
| **Biochemical variables** | **Total population**  **(n=112)** | **Normal**  **(n=27)** | **Osteopenia**  **(n=49)** | **Osteoporosis**  **(n=36)** | **p-value** | **TBS > 1.31**  **(n=41)** | **TBS = 1.31-1.23 (n=23)** | **TBS < 1.23**  **(n=45)** | **p-value** |
| **Total protein, g/L** | 73 (70-76) | 72 [71-75.75] | 73 [70-74.25] | 73 [70.5-76.5] | 0.372 | 72 [70.5-75] | 72 [70.25-74] | 73 [70-76] | 0.579 |
| **Chloride, mEq/L** | 102 (101-104) | 103 [101.5-105] | 102 [101-104] | 102 [101-104] | 0.357 | 102 [101-104] | 102 [100.5-103] | 102 [101-105] | 0.98 |
| **Venous pH** | 7.34 (7.31-7.37) | 7.35 [7.32-7.37] | 7.34 [7.32-7.37] | 7.32 [7.3-7.36] | 0.211 | 7.35 [7.32-7.37] | 7.33 [7.3-7.37] | 7.33 [7.3-7.36] | 0.666 |
| **Venous pCO_2_, mmHg** | 50.45 (44.18-54.10) | 46.3 [42.8-51.3] | 50.4 [44.1-53] | 51.4 [48.38-57.68] | **0.012** | 50.5 [43.1-54.4] | 50.3 [48.6-54.1] | 50.4 [44.2-52.9] | 0.448 |
| **Magnesium, mg/dL** | 2 (1.9-2.2) | 2 [1.9-2.1] | 2 [1.9-2.12] | 2.1 [2-2.3] | 0.786 | 2 [1.9-2.12] | 2.1 [1.9-2.15] | 2.1 [1.9-2.3] | 0.844 |
| **Phosphorus, mg/dL** | 3.1 (2.8-3.5) | 3.1 [2.85-3.45] | 3.1 [2.7-3.5] | 3.35 [2.98-3.5] | 0.799 | 3.1 [2.7-3.5] | 3.4 [3.05-3.6] | 3.1 [2.8-3.5] | 0.233 |
| **Testosterone, nmol/L** | 3.375 (2.6-4.36) | 4.03 [2.62-4.71] | 3.24 [2.6-3.86] | 3.68 [2.61-4.7] | 0.394 | 3.8 [2.8-5.09] | 3.55 [2.57-4.36] | 3.21 [2.37-4.06] | 0.057 |
| **Estradiol, pg/mL** | 27 (20-33) | 30 [22.5-36] | 27 [20-33] | 25 [18-31.5] | 0.282 | 30 [22-34] | 30 [15.25-34] | 24 [19.5-30] | 0.367 |
| **Radiological variables** | **Total population**  **(n=112)** | **Normal**  **(n=27)** | **Osteopenia**  **(n=49)** | **Osteoporosis**  **(n=36)** | **p-value** | **Normal**  **(n=41)** | **Partially degraded**  **(n=23)** | **Degraded**  **(n=45)** | **p-value** |
| **Lumbar spine Z-score, SD** | -0.67 (-1.76-0.69) | 0.56 [-0.31-2.15] | -0.66 [-1.78-0.71] | -1.69 [-2.08--0.91] | **<0.001** | 0.06 [-1.28-0.98] | -0.9 [-1.95-0.34] | -1.05 [-1.78--0.08] | 0.136 |
| **Femoral neck Z-score, SD** | -0.55 (-1.03-0.34) | 0.7 [0.29-0.93] | -0.39 [-0.86-0.07] | -1.25 [-1.49--0.78] | **<0.001** | 0.04 [-1.02-0.51] | -0.7 [-0.9-0.22] | -0.76 [-1.24--0.01] | 0.159 |
| **Total hip Z-score, SD** | -0.315 (-1.09-0.43) | 0.77 [0.33-1.28] | -0.27 [-0.71-0.32] | -1.15 [-1.48--0.72] | **<0.001** | 0.04 [-1.02-0.51] | -0.7 [-0.9-0.22] | -0.76 [-1.24--0.01] | 0.159 |
| **TBS T-score, SD** | -1.7 (-2.5--0.97) | -1.55 [-2.5--0.85] | -1.6 [-2.6--1.1] | -2 [-2.4--1.2] | 0.275 | -0.8 [-1.1-0.1] | -1.6 [-1.9--1.5] | -2.7 [-3.08--2.32] | **<0.001** |
| **TBS Z-score, SD** | 0 (-0.88-0.78) | 0.3 [-1-1] | 0 [-0.8-0.83] | -0.2 [-0.75-0.4] | 0.45 | 1 [0.6-1.5] | 0 [-0.2-0.1] | -1.1 [-1.55--0.7] | **<0.001** |

**Quantitative variables** are expressed as median (IQR), and **qualitative variables** as number (%).

**Abbreviations: BMD:** bone mineral density; **TBS:** trabecular bone score; **SD:** standard deviation; **CKD:** chronic kidney disease; **RC:** radical cystectomy.

**Definitions:** BMD according to WHO criteria: normal (T-score ≥ -1 SD), osteopenia (T-score -1 to -2.5 SD), osteoporosis (T-score ≤ -2.5 SD).

**Note: Statistically significant p-values are shown in bold**
