# Supplementary material for: Fracture risk assessment in patients with ileal urinary diversion after radical cystectomy: a comprehensive evaluation integrating bone mineral density, trabecular bone score, and FRAX®
Source: Arch Osteoporos. 2026 Mar 11;21(1):50. doi: 10.1007/s11657-026-01685-x (PMC12979281; doi:10.1007/s11657-026-01685-x)
Supplement: Supplementary file 6 — (DOCX 26.6 KB) [file 11657_2026_1685_MOESM6_ESM.docx]

**Supplementary Table S3.** Comparison of clinical, biochemical, bone assessment variables, and fracture risk between patients with and without metabolic acidosis.

| **Clinical variables** | **Without Metabolic Acidosis** | **With Metabolic acidosis** | **p-value** |
| --- | --- | --- | --- |
| **N (%)** | 96 (85.71%) | 16 (14.29%) |  |
| **Age, years** | 71.5 [64-77] | 68 [67-74] | 0.674 |
| **Type of urinary diversion, n (%)** |  |  | 0.5 |
| **Bricker** | 88 (91.7 %) | 16 (100.0 %) |  |
| **Neobladder** | 8 (8.3 %) | 0 (0%) |  |
| **Follow-up time, months** | 36.56 [14.24-85.96] | 29.77 [15.39-85.6] | 0.944 |
| **BMI, Kg/m^2^** | 28.34 [25.95-31.87] | 27 [25.24-31.58] | 0.36 |
| **Dietary calcium intake, mg** | 650 [500-800] | 625 [487.5-725] | 0.612 |
| **History of bone fracture, n (%)** | 30 (31.2 %) | 3 (18.8 %) | 0.472 |
| **Family history of osteoporotic fracture, n (%)** | 19 (19.8 %) | 4 (25.0 %) | 0.886 |
| **Current smoker, n (%)** | 22 (22.9 %) | 6 (37.5 %) | 0.212 |
| **Glucocorticoid therapy, n (%)** | 3 (3.1 %) | 0 (0.0 %) | 1 |
| **Rheumatoid arthritis, n (%)** | 0 (0 %) | 1 (6.2 %) | 0.305 |
| **Secondary osteoporosis, n (%)** | 4 (4.2 %) | 0 (0 %) | 0.917 |
| **Alcohol >3 U/d, n (%)** | 9 (9.4 %) | 1 (6.2 %) | 1 |
| **DM, n (%)** | 24 (25.0 %) | 5 (31.2 %) | 0.597 |
| **Anemia, n (%)** | 19 (19.8 %) | 11 (68.8 %) | **<0.001** |
| **CKD prior to RC, n (%)** | 11 (11.5 %) | 4 (25.0 %) | 0.282 |
| **CKD after RC, n (%)** | 45 (46.9 %) | 16 (100.0 %) | **<0.001** |
| **Bicarbonate therapy, n (%)** | 1 (1.0 %) | 3 (18.8 %) | 0.005 |
| **Vitamin D therapy, n (%)** | 14 (14.6 %) | 1 (6.2 %) | 0.61 |
| **Vertebral fracture, n (%)** | 9 (9.4 %) | 2 (12.5 %) | 1 |
| **Fracture after RC, n (%)** | 3 (3.1 %) | 0 (0 %) | 0.773 |
| **Biochemical variables** | **Without Metabolic Acidosis** | **With Metabolic acidosis** | p-value |
| **Hemoglobin, g/L** | 145 [134.75-154.25] | 116 [107.75-133] | **<0.001** |
| **Albumin, g/L** | 45.05 [42.15-47.02] | 42.25 [41.38-44.33] | **0.029** |
| **Total protein, g/L** | 72 [70-75] | 74 [71.5-76] | 0.364 |
| **Calcium, mg/dL** | 9.6 [9.4-9.9] | 9.4 [9.2-9.6] | **0.01** |
| **25 (OH)D, ng/mL** | 18.15 [12.6-24.52] | 13.4 [10.46-15.85] | **0.034** |
| **PTH, pg/mL** | 63 [45.5-82] | 72 [58.75-152.25] | **0.0489** |
| **ALP, U/L** | 81 [62-97.25] | 84 [72.25-107.25] | 0.401 |
| **Chloride, mEq/L** | 102 [101-103] | 106 [104.75-107.5] | **<0.001** |
| **Magnesium, mg/dL** | 2.05 [1.9-2.2] | 2 [1.9-2.05] | 0.382 |
| **Phosphorus, mg/dL** | 3.1 [2.7-3.5] | 3.5 [3-3.75] | **0.0496** |
| **CTX, ng/mL** | 0.38 [0.24-0.48] | 0.64 [0.32-0.84] | **0.0048** |
| **IGF-1, ng/mL** | 131 [110-171] | 130 [98.5-195.25] | 0.775 |
| **Venous pH** | 7.35 [7.31-7.37] | 7.3 [7.26-7.34] | **0.0023** |
| **Venous pCO_2_, mmHg** | 51.2 [46.3-55.15] | 41.1 [39.1-44.05] | **<0.001** |
| **Venous bicarbonate, mEq/L** | 27.1 [24.7-28.83] | 20.5 [18.7-21.63] | **<0.001** |
| **Venous base excess, mmol/L** | 0.6 [-0.7-2.22] | -5.45 [-7.92--4.02] | **<0.001** |
| **Creatinine, mg/dL** | 1.25 [1.03-1.44] | 2 [1.68-3.28] | **<0.001** |
| **eGFR, mL/min/m2** | 59.5 [47.75-74] | 34.5 [17-41.5] | **<0.001** |
| **Testosterone, nmol/L** | 3.61 [2.68-4.51] | 2.46 [1.94-3.34] | **0.0395** |
| **Estradiol, pg/mL** | 26 [19-33] | 28.5 [22.75-33] | 0.501 |
| **Radiological variables** | **Without Metabolic Acidosis** | **With Metabolic acidosis** | **p-value** |
| **Lumbar spine BMD, g/cm^2^** | 1.13 [0.97-1.27] | 1.15 [1.03-1.37] | 0.399 |
| **Lumbar spine T-score, SD** | -0.98 [-2.24-0.16] | -0.8 [-1.82-1.12] | 0.413 |
| **Lumbar spine Z-score, SD** | -0.68 [-1.77-0.68] | -0.56 [-1.6-2.7] | 0.308 |
| **Femoral neck BMD, g/cm^2^** | 0.87 [0.73-0.94] | 0.89 [0.78-0.96] | 0.919 |
| **Femoral neck T-score, SD** | -1.58 [-2.57--0.84] | -1.31 [-2.24--0.86] | 0.971 |
| **Femoral neck Z-score, SD** | -0.64 [-1-0.17] | -0.01 [-1.27-0.37] | 0.83 |
| **Total hip BMD, g/cm^2^** | 0.92 [0.8-1.02] | 0.95 [0.84-1.03] | 0.882 |
| **Total hip T-score, SD** | -1.27 [-2.2--0.43] | -1.08 [-1.47--0.47] | 0.829 |
| **Total hip Z-score, SD** | -0.36 [-1.1-0.43] | 0.03 [-0.78-0.42] | 0.65 |
| **TBS L1-L4** | 1.27 [1.18-1.35] | 1.26 [1.18-1.35] | 0.724 |
| **TBS T-score, SD** | -1.7 [-2.5--0.9] | -2 [-2.6--1.15] | 0.616 |
| **TBS Z-score, SD** | 0 [-0.8-0.9] | 0.1 [-1.1-0.5] | 0.392 |
| **Risk of fracture** | **Without Metabolic Acidosis** | **With Metabolic acidosis** | p-value |
| **FRAX-MOF without BMD** | 4 [2.7-6.23] | 3.8 [2.7-4.73] | 0.705 |
| **FRAX-MOF with BMD** | 5.35 [2.9-7.73] | 4.1 [3.18-8.4] | 0.987 |
| **FRAX-MOF adjusted with TBS** | 6.1 [3.4-9.5] | 5.1 [3.08-9.65] | 0.891 |
| **FRAX-HIP without BMD** | 1.5 [0.67-3] | 1.05 [0.78-2.1] | 0.612 |
| **FRAX-HIP with BMD** | 1.8 [0.9-4.55] | 1.6 [0.88-4.15] | 0.97 |
| **FRAX-HIP adjusted with TBS** | 2 [1-5.2] | 1.75 [0.98-4.12] | 0.761 |
| **BMD categories** | **Without Metabolic Acidosis** | **With Metabolic acidosis** | P = 0.791 |
| **Osteoporosis, n (%)** | 32 (33.3%) | 4 (25%) |  |
| **Osteopenia, n (%)** | 41 (42.7%) | 8 (50%) |  |
| **Normal BMD, n (%)** | 23 (24%) | 4 (25%) |  |
| **TBS categories** | **Without Metabolic Acidosis** | **With Metabolic acidosis** | P = 0.898 |
| **TBS degraded, n (%)** | 38 (39.8%) | 7 (43.8%) |  |
| **TBS partially degraded, n (%)** | 20 (20.8%) | 3 (18.8%) |  |
| **TBS normal, n (%)** | 35 (36.5%) | 6 (37.5%) |  |
| **TBS excluded due to artifacts, n (%)** | 3 (3.1%) | 0 (0%) |  |

**Quantitative variables** are expressed as median (IQR), and **qualitative variables** as number (%).

**Abbreviations: BMD:** bone mineral density; **TBS:** trabecular bone score; **BMI:** body mass index; **DM:** diabetes mellitus; **CKD:** chronic kidney disease; **RC:** radical cystectomy; **25(OH)D:** 25-hydroxyvitamin D; **PTH:** parathyroid hormone; **ALP:** alkaline phosphatase; **CTX:** C-terminal telopeptide of type 1 collagen; **IGF-1:** insulin-like growth factor 1; **pCO₂:** venous partial pressure of CO₂; **eGFR:** estimated glomerular filtration rate; **FRAX:** Fracture Risk Assessment Tool; **FRAX-MOF:** The 10-year risks of major osteoporotic fracture; **FRAX-HIP:** The 10-year risks of hip fracture.

**Definitions:** Metabolic acidosis defined as venous serum bicarbonate <22 mEq/L; Anemia according to WHO criteria (<13 g/dL in men); CKD defined by eGFR <60 mL/min/1.73 m²; BMD according to WHO criteria: normal (T-score ≥ -1 SD), osteopenia (T-score -1 to -2.5 SD), osteoporosis (T-score ≤ -2.5 SD).

**Note: Statistically significant p-values are shown in bold**
