# Supplementary material for: Fracture risk assessment in patients with ileal urinary diversion after radical cystectomy: a comprehensive evaluation integrating bone mineral density, trabecular bone score, and FRAX®
Source: Arch Osteoporos. 2026 Mar 11;21(1):50. doi: 10.1007/s11657-026-01685-x (PMC12979281; doi:10.1007/s11657-026-01685-x)
Supplement: Supplementary file 7 — (DOCX 18.6 KB) [file 11657_2026_1685_MOESM7_ESM.docx]

**Supplementary Table S2.** Characteristics of vertebral fractures identified by vertebral fracture assessment (VFA) in ten patients: type, grade, location, and bone parameters (L2–L4 T-score, L1–L4 TBS).

| **Patient** | **Single or multiple fracture** | **Type of deformity** | **Grade** | **Location** | **T-score (L2–L4)** | **TBS**  **(L1–L4)** |
| --- | --- | --- | --- | --- | --- | --- |
| 1 | multiple | wedge  wedge  biconcave  biconcave  crush | moderate  severe  moderate  severe  moderate | T10, T11  T8, L2  L3  T12  T7 | -2.56 | 0.949 |
| 2 | multiple | wedge | moderate | T11, T12 | -0.53 | 1.206 |
| 3 | single | wedge | moderate | L1 | -3.68 | 1.014 |
| 4 | single | biconcave | moderate | T11 | 0.27 | 1.180 |
| 5 | multiple | wedge  crush | moderate  moderate | T8  T7 | -2.185 | 1.301 |
| 6 | single | wedge | severe | L3 | -1.26 | 1.360 |
| 7 | single | wedge | severe | T10 | 0.31 | 1.292 |
| 8 | single | biconcave | severe | T10 | -3.96 | 1.091 |
| 9 | single | biconcave | moderate | T8 | -3.58 | 1.239 |
| 10 | single | wedge | severe | T12 | -2.22 | 1.000 |
| 11 | multiple | wedge | moderate | T7, T8, T9 | -2.60 | 1.284 |

**Abbreviations:** **TBS:** trabecular bone score; Vertebrate Types: (**T**: Thoracic; **L**: Lumbar)

**Definitions:** Vertebral fractures were defined according to the Genant semiquantitative method, classifying both deformity type (wedge, biconcave, or crush) and grade based on vertebral height loss: mild (20–25%), moderate (25–40%), or severe (>40%).
